# Mixed Acinar-Endocrine Carcinoma (MAEC) Arising in Duodenal Pancreatic Heterotopia

**DOI:** 10.1155/2019/1713546

**Published:** 2019-09-02

**Authors:** Phoenix D. Bell, Tom C. DeRoche, Aaron R. Huber

**Affiliations:** ^1^Department of Pathology, University of Rochester Medical Center, Rochester, NY, USA; ^2^Department of Pathology, Kaiser Permanente, Portland, OR, USA

## Abstract

Mixed acinar-endocrine carcinoma (MAEC) of the pancreas is a rare neoplasm, consisting of at least 25%–30% of acinar and neuroendocrine populations. Patients are often middle-aged and present with nonspecific symptoms. Imaging typically reveals a solid lesion in the pancreatic head. Management involves surgical resection and the overall prognosis is variable. Here, we present a case of a 48-year-old male who presented with a MAEC arising from duodenal pancreatic heterotopia. This is the one of the first cases, with histologic evidence, of MAEC arising from pancreatic heterotopia.

## 1. Introduction

Pancreatic tumors are rare and may arise from either endocrine or exocrine components. Neuroendocrine cells make up the endocrine component, while ductal and acinar cells make up the exocrine component. The majority of pancreatic malignancies are ductal adenocarcinomas (>75%), followed by neuroendocrine tumors (NET) (7%), and acinar cell carcinomas (ACC) (1%) [1, 2]. Up to one third of ACC contain small numbers of neuroendocrine cells, usually in small clusters [3, 4]. Rarely, ACC contain a significant neuroendocrine population. Pancreatic tumors with at least 25%–30% of both acinar and neuroendocrine cell types, are classified as mixed acinar-endocrine carcinomas (MAEC) [5, 19]. MAEC is a rare pancreatic tumor that almost always arises in the pancreatic head of middle-aged adults, and patients often present with nonspecific symptoms [1, 2]. Extremely rare cases of MAEC arising from pancreatic heterotopia have been reported in the literature [6–8].

## 2. Case Presentation

**Figure 1:**
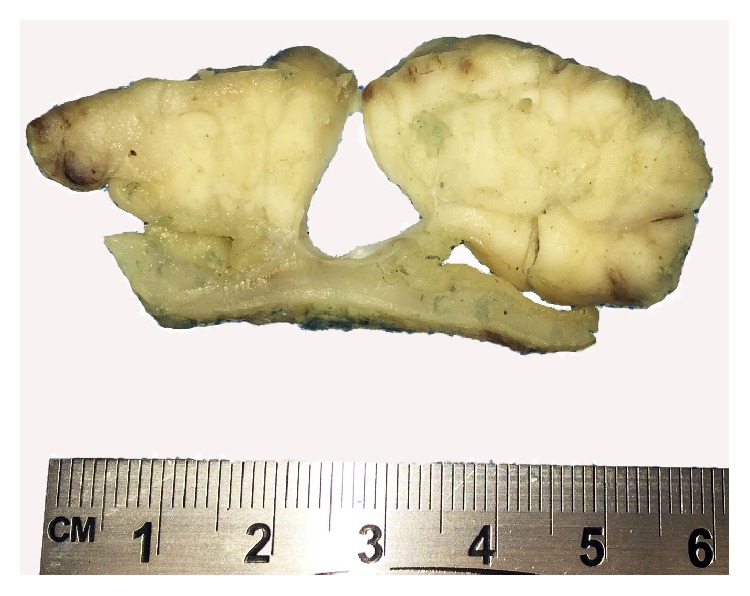
Large fungating mass centered in the duodenum.

A 48-year-old male with past medical history of asthma, chronic pain, and obesity presented with progressive fatigue and epigastric discomfort for approximately six to eight weeks. A complete blood count revealed a hemoglobin level of 5.2 g/dL and esophagogastroduodenoscopy (EGD) demonstrated a large ulcerated mass in the duodenum, occupying approximately 90% of the circumference of the duodenal wall. The mass extended from the apex of the duodenal bulb to the major papilla; however, the major papilla was unremarkable. A follow up computed tomography scan showed a 6.0 × 5.5 × 5.0 cm hypoattenuating mass involving the mesenteric aspect of the second portion of the duodenum, which approached the proximal third segment of the duodenum. Additionally, the mass appeared to involve the pancreatic head and uncinate process; however, no hepatobiliary or pancreatic ductal obstruction was noted. Biopsy revealed a poorly differentiated carcinoma with neuroendocrine features. The patient underwent a pancreaticoduodenectomy (Whipple procedure) which showed a 10.2 × 8 × 2.7 cm pink-tan, lobulated, fungating mass with central necrosis within the duodenum. The mass was centered in the lumen of the duodenum with a well-demarcated (pushing) front of macroscopic invasion into the pancreas (Figure 1).

**Figure 2:**
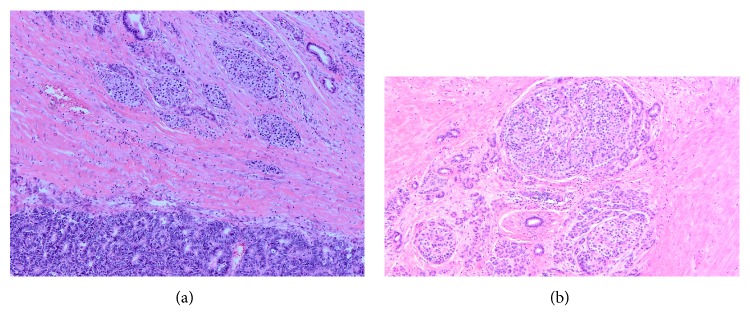
(a and b) Duodenal tumor adjacent to pancreatic heterotopia composed of pancreatic acini, islets of Langerhans, and ducts (original magnification ×100 and ×200).

**Figure 3:**
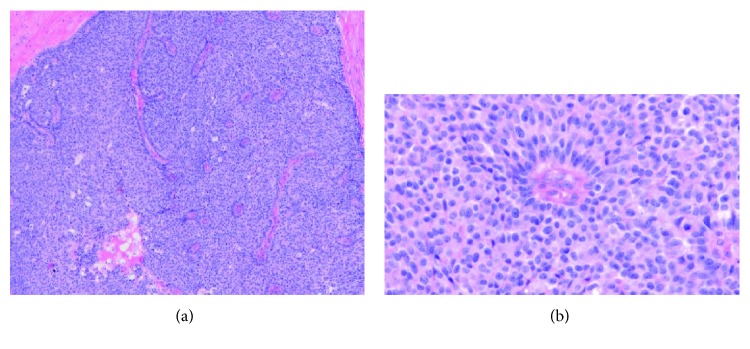
(a and b) Neuroendocrine cell component (original magnification ×100 and ×400).

**Figure 4:**
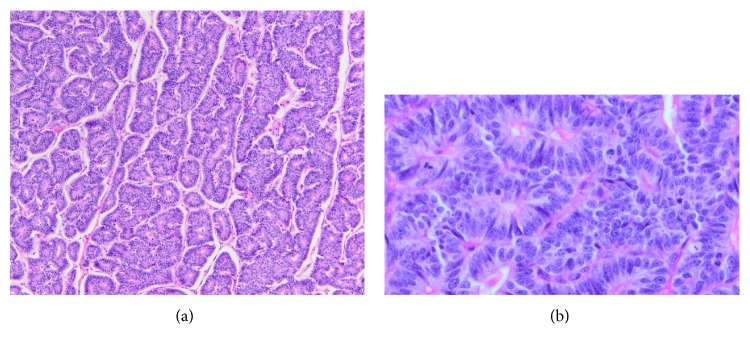
(a and b) Acinar cell component (original magnification ×100 and ×400).

**Figure 5:**
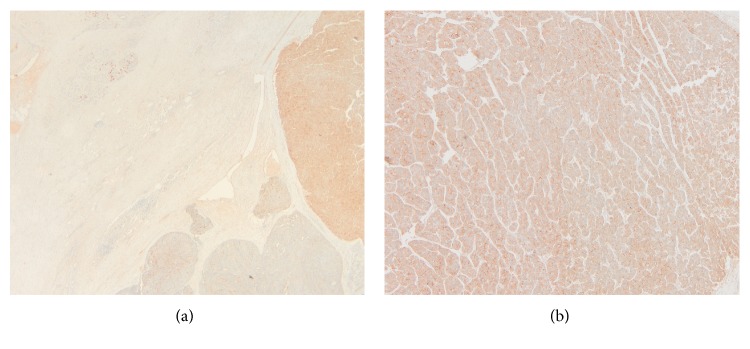
(a and b) Trypsin positivity in the acinar component (original magnification ×100 and ×200).

**Figure 6:**
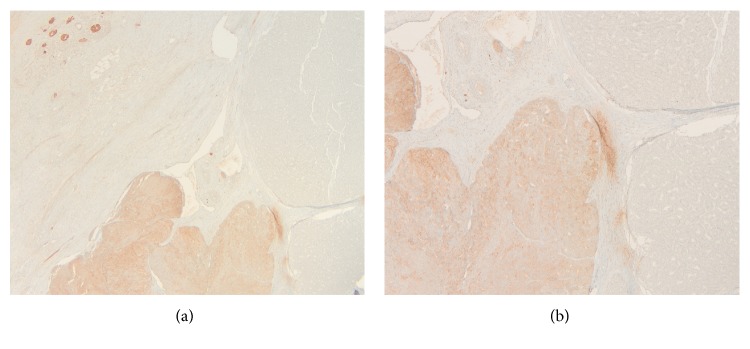
(a and b) Synaptophysin in the neuroendocrine component (original magnification ×100 and ×200).

**Figure 7:**
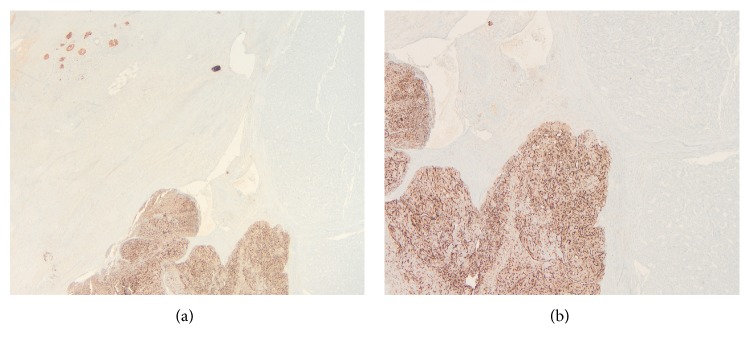
(a and b) Chromogranin in the neuroendocrine component (original magnification ×100 and ×200).

Microscopic examination of the lesion showed a neoplasm arranged primarily in lobules with prominent acinar formation. There were focal areas where the tumor was arranged in solid sheets. The neoplasm was located in the duodenum adjacent to an area of pancreatic heterotopia (Figures 2(a) and 2(b)). Some of the cells had moderate to abundant eosinophilic cytoplasm and nuclei with smooth contours and granular chromatin. Some other areas demonstrated pseudorosettes (Figures 3(a) and 3(b)), while others showed a typical acinar pattern with cells that had moderate to abundant granular eosinophilic cytoplasm, nuclei with open chromatin and prominent nucleoli, and increased mitotic figures (40/10 hpf) (Figures 4(a) and 4(b)). Immunohistochemical analysis revealed an acinar population, which stained positively for trypsin (Figures 5(a) and 5(b)) and BCL-10, as well as a neuroendocrine population that stained positively for synaptophysin (Figures 6(a) and 6(b)) and chromogranin (Figures 7(a) and 7(b)). Both acinar and neuroendocrine cell populations made up >25%–30% of the lesion, thus the patient was diagnosed with a MAEC.

Following surgical resection, the postoperative course was complicated by several bacterial infections, pulmonary abscesses, and liver metastases. Unfortunately, the patient died approximately six months after initial presentation.

## 3. Discussion

MAEC is a rare pancreatic tumor, consisting of at least 25%–30% acinar and neuroendocrine cells [5, 19]. These tumors most commonly arise in the head of the pancreas [1, 9] and are almost always nonfunctional [3, 10]. Patients present in middle-age [1, 10] with nonspecific symptoms including abdominal pain and weight loss [2, 3]. As there is no way to radiographically differentiate MAEC from other solid pancreatic head neoplasms [11, 12], definitive diagnosis relies upon histopathologic analysis.

Patients undergo initial evaluation with fine-needle aspiration, yet cytologic evaluation of MAEC can be extremely difficult. In some circumstances, it is easy to identify cells in an acinar formation that have granular cytoplasm and prominent nucleoli, suggesting ACC [13]. Similarly, cases that have cells with abundant cytoplasm, eccentric nuclei, and evenly distributed chromatin classically describe features of an endocrine tumor [14]. Another clue to the diagnosis of an ACC component is a high mitotic rate that is much higher than one might expect in a NET, as seen in this case. However, when there is a monotonous population of large cells, some single and others in small clusters, perhaps hinting at acinar formation, and only some cells have prominent nucleoli, the diagnosis is less obvious [15]. Further, diagnosis may be complicated by sampling error in which only the acinar or only the neuroendocrine population is taken or if there is insufficient cellular material available for diagnosis/IHC studies [13, 15]. Although NET are more prevalent, reports of MAEC are increasing, likely due to increased awareness and application of IHC [3].

Definitive diagnosis of MAEC relies upon examination of the resection specimen to show the necessary proportion of acinar and neuroendocrine cells. There are two main patterns of MAEC described in the literature, which should be considered when the differential includes ACC, NET, and MAEC. These patterns include (1) two morphologically distinct and isolated populations of cells or (2) two intermingled populations of cells [4, 10, 15]. In certain cases, the distinction between acinar and neuroendocrine populations may be unclear. For example, in our case, rosette structures were present suggesting NET; however, the prominent nucleoli and elevated mitotic rate were more characteristic of ACC, thus we pursued immunohistochemical analysis. When using stains, it is important to remember that up to one-third of ACC may express neuroendocrine markers, albeit they are usually scattered cells [1, 3, 10]. The acinar cells stain positively for trypsin, chymotrypsin, lipase, and PAS, while neuroendocrine cells stain positively for chromogranin and synaptophysin.

There are few reports of MAEC published in the literature, and even fewer published arising in pancreatic heterotopia. Moncur et al. [6] described an autopsy case from a patient with a tumor arising in the ampulla of Vater with metastasis to the liver, lungs, and vertebrae. The cells stained for amylase and synaptophysin, and weakly for trypsin; however, no pancreatic heterotopia was found histologically. They suggest the tumor most likely arose from heterotopia since a pancreatic mass was not grossly identified. Kusafuka et al. [7] detail a MAEC arising from the stomach, yet there is no microscopic suggestion of pancreatic heterotopia. Despite the lack of microscopic evidence, they suggest the neoplasm arose in heterotopia or may have differentiated from pluripotent stem cells. Steel et al. [8] report MAEC of the liver, which they hypothesized metastasized from the pancreas or, less likely, arose from ectopic pancreatic tissue in the liver. A 14-month follow-up did not demonstrate a pancreatic mass, thus the authors conclude the lesion arose from heterotopia; however, the microscopic images do not reflect this finding. As detailed, there are few studies published on MAEC arising from heterotopia, and of those, histologic evidence is speculative or scant.

The etiology and behavior of MAEC are not well understood. Literature documents fairly poor prognosis, similar to that of ACC, and suggests that tumors with increased numbers of neuroendocrine cells have a better prognosis [1]. Some authors believe this supports MAEC as a subtype of ACC [3]. Currently, the two main hypotheses suggest (1) that the neuroendocrine and acinar cell differentiation arises from pluripotent stem cells [3] or (2) differentiation is triggered by somatic mutations [15]. Interestingly, ACC is more common in males; however, a gender predilection is not as evident for MAEC with a split between studies favoring male predilection [1, 10] and others supporting female predilection [3, 5].

The molecular characteristics of pancreatic NET, neuroendocrinecarcinoma (NEC), and ACC have been well characterized. Pancreatic NET and NEC have an average of 16 somatic mutations. The major genes involved in these somatic mutations are *MEN1, ATRX, DAXX, TSC2, PTEN, Rb, and TP53* [17]. ACC has an average of 131 somatic mutations. The major genes involved are* SMAD4, JAK1, BRAF, RB1, TP53, APC, ARID1A, GNAS, MLL3, and PTEN* [17]. On the other hand, the literature analyzing mutations in MAEC is sparse, likely due to their rarity. Takan et al. [18] have described a single case of TP53 and KRAS mutations in a case of pancreatic mixed acinar-neuroendocrine-ductal carcinoma in both the acinar/neuroendocrine and ductal components, suggesting that each component arose from a single tumor clone. This supports the theory that a single tumor clone may lead to divergent differentiation and mixed tumors [18].

Since the pathogenesis is not fully understood, there is no standardized treatment available for MAEC. With comparable aggressiveness to that of ACC [3, 9, 10], it is generally accepted to pursue surgical resection if the tumor is operable. Some studies have documented S-1 chemotherapy response for MAEC cases that were initially diagnosed as pancreatic adenocarcinoma or ACC. Seino et al. [12] suggest this type of chemotherapy treats the acinar cell population, yet leaves the neuroendocrine component available to proliferate. Similarly, Yokode et al. [4] debate whether S-1 chemotherapy helps treat MAEC or whether the drug induces neuroendocrine differentiation. This is also similar to the report from Kanemasa et al. [16] which showed an increase in neuroendocrine cells following S-1 treatment. Alternatively, Hara et al. [9] reported a case of MAEC which metastasized to the liver, which was treated by arterial catheter embolization and pancreatectomy, proposing that this type of embolization may be useful for liver metastasis. Overall, surgery is the treatment of choice and the efficacy of other therapies, such as chemotherapy and embolization, require additional investigation.

In conclusion, we present a rare case of MAEC arising from pancreatic heterotopia in the duodenum. Owing to the morphologic overlap between MAEC, ACC, and NET, it is important to consider these differentials, especially when tissue is limited on FNA or cell block. ACC are much more aggressive than NET of the pancreas, and it is suspected that MAEC have a prognosis similar to that of ACC. Thus, it is important to accurately diagnose these lesions as the management may differ. With increased recognition of MAEC, especially with improved IHC, it is likely that additional studies will be completed which will contribute to improved management of these neoplasms.
